# A Survey of Referrals to Psychiatric Intensive Care Unit (PICU): Patient Characteristics and Outcome

**DOI:** 10.1192/bjo.2024.505

**Published:** 2024-08-01

**Authors:** Shantala Satisha, Emily Pettifor

**Affiliations:** Willow Suite Psychiatric Intensive Care Unit, Littlebrook Hospital, Dartford, United Kingdom

## Abstract

**Aims:**

The project aims to evaluate the referrals from North Kent for admission to our PICU from April to November 2021.

Hypothesis:

There are very few surveys of PICU referrals. We expect more referrals for younger men with psychotic illnesses and comorbid diagnoses; to be for aggressive behaviours; and most will be admitted to acute wards with ongoing support from the PICU liaison team.

**Background:**

Our PICU services in the trust include one 12-bedded male PICU, 5 contracted female PICU beds and the PICU liaison service. PICU liaison team ‘gatekeep’ the PICU beds for patients meeting the admission criteria and supporting the other referrals’ admissions to non-PICU acute beds by working closely with the staff and patients on these wards.

**Methods:**

Data was collected for all referrals for PICU admission made to one of the three PICU Liaison practitioners in North Kent from April to November 2021, recording the demographics, clinical information and outcomes.

**Results:**

There were 126 referrals in this time period, of which 68% were males. 38% were aged 18–30 and 25% were 31–40 years old.

43% of referrals were from inpatient acute wards, 21% from community, 21% from other settings and 7% from Places of Safety. 75% of the referrals were detained under the Mental Health Act.

The primary diagnosis was Schizophrenia in 25%, Bipolar Affective Disorder in 25%, Schizoaffective Disorder in 13%, Personality Disorders and Substance misuse related disorders were 7% each. 32% of the referrals had a comorbid diagnosis; 43% of which was substance use related, 23% had personality disorder and 34% had other conditions including neurodevelopmental disorders.

42% had previous admissions to PICU and 52% had forensic history.

Reason for referral was aggression in 76%; 10% did not have any indications for PICU and 18% was for current or recent prison stay.

30% of the referrals were admitted to PICU and 58% were either admitted to or remained on the acute wards with support from PICU Liaison Team. While 5% were diverted to the forensic pathway, 7% remained in the community.

**Conclusion:**

In conclusion, the data shows patients referred for PICU admission were more likely to be young men with aggressive behaviour and a primary psychotic illness, using illicit substances. Most referrals came from the inpatient wards as is to be expected. They were also more likely to have previous PICU admissions and a significant forensic history.

